# Epithelial–Mesenchymal Transition in Kidney Tubular Epithelial Cells Induced by Globotriaosylsphingosine and Globotriaosylceramide

**DOI:** 10.1371/journal.pone.0136442

**Published:** 2015-08-20

**Authors:** Yeo Jin Jeon, Namhee Jung, Joo-Won Park, Hae-Young Park, Sung-Chul Jung

**Affiliations:** Department of Biochemistry, School of Medicine, Ewha Womans University, Seoul, South Korea; Seoul National University, REPUBLIC OF KOREA

## Abstract

Fabry disease is a lysosomal storage disorder caused by deficiency of alpha-galactosidase A (α-gal A), which results in the deposition of globotriaosylceramide (Gb3) in the vascular endothelium. Globotriaosylsphingosine (lyso-Gb3), a deacylated Gb3, is also increased in the plasma of patients with Fabry disease. Renal fibrosis is a key feature of advanced Fabry disease patients. Therefore, we evaluated the association of Gb3 and lyso-Gb3 accumulation and the epithelial–mesenchymal transition (EMT) on tubular epithelial cells of the kidney. In HK2 cells, exogenous treatments of Gb3 and lyso-Gb3 increased the expression of TGF-β, EMT markers (N-cadherin and α-SMA), and phosphorylation of PI3K/AKT, and decreased the expression of E-cadherin. Lyso-Gb3, rather than Gb3, strongly induced EMT in HK2 cells. In the mouse renal mesangial cell line, SV40 MES 13 cells, Gb3 strongly induced phenotype changes. The EMT induced by Gb3 was inhibited by enzyme α-gal A treatment, but EMT induced by lyso-Gb3 was not abrogated by enzyme treatment. However, TGF-β receptor inhibitor (TRI, SB525334) inhibited the activation of TGF-β and EMT markers in HK2 cells with Gb3 and lyso-Gb3 treatments. This study suggested that increased plasma lyso-Gb3 has a crucial role in the development of renal fibrosis through the cell-specific induction of the EMT in Fabry disease, and that TRI treatment, alongside enzyme replacement therapy, could be a potential therapeutic option for patients with Fabry disease.

## Introduction

Fabry disease (FD) is an X-linked lysosomal storage disorder caused by deficient activity of the lysosomal enzyme alpha-galactosidase A (α-gal A) [1,2]. A loss of enzymatic α-gal A results in the progressive accumulation of globotriaosylceramide (Gb3) in lysosomes, extracellular spaces, and other cellular compartments, and leads to disease manifestation and tissue injury [3]. Patients develop painful neuropathy and vascular occlusions that progressively lead to cardiovascular, cerebrovascular, and renal dysfunction, and early death [1,2]. High serum concentration of globotriaosylsphingosine (lyso-Gb3), indicating the presence of deacylated Gb3, has been observed in patients with FD [4].

Kidney fibrosis is a key feature of FD and is supposed to play a major role in FD nephropathy [5]. Fibrosis is characterized by deposition of extracellular matrix (ECM) components, such as collagens, fibronectin, elastin, tenascin, and other matrix molecules [6–8]. The activation of interstitial fibroblasts to cause myofibroblasts that secrete ECM components is associated with renal fibrosis. The expression of α-smooth muscle actin (α-SMA), a specific marker of myofibroblasts, is not activated in all fibroblasts, but increases during endothelial–mesenchymal transition or epithelial–mesenchymal transition (EMT) [9]. During tissue injury, the EMT is a major mechanism promoting the development of renal interstitial fibrosis. Recent studies have suggested that, in mouse models with renal fibrosis, myofibroblasts can be generated from renal tubular epithelial cells that undergo the EMT [10,11]. Kidney injury is associated with inflammatory cells, which can give rise to EMT using various growth and differentiation factors, such as TGF-β, receptor tyrosine kinase, Integrin, Wnt, and Notch proteins [12], and under the influence of these, resident fibroblasts and tubular epithelial cells produce basement membrane-degrading enzymes. The induction of the EMT by TGF-β was first recognized in cell culture and has received much attention as a key process that is active during embryonic development, cancer progression, and fibrosis [13].

Recent studies reported that lyso-Gb3 promotes proliferation of vascular smooth muscle cells [14,15], which suggests that lyso-Gb3 has a role in the pathogenesis of FD, and acts as a secondary mediator of glomerular injury and fibrosis in cultured human podocytes. Podocyte injury has been strongly suggested to have a pivotal role in the development and progression of Fabry nephropathy [5,15]. However, interstitial fibrosis and tubular atrophy are also found in patients with FD in addition to glomerulosclerosis [16,17]. We hypothesized that, in addition to podocyte injury, the potential direct effects of Gb3 or lyso-Gb3 on tubular cells may lead to tubular cell activation and interstitial fibrosis, which can contribute to disease progression [5]. In this study, we used human proximal renal tubular epithelial cells (HK2) and mouse glomerular mesangial cells (SV40 MES 13) as cellular models. We observed that the phenotype changes depended on Gb3 or lyso-Gb3 in these cells. We focused on the EMT induction by Gb3 or lyso-Gb3 on tubular cells, rather than podocytes. We also explored potential therapeutic approaches by using a recombinant lysosomal enzyme and anti-EMT drugs, which are considered in the treatment of these conditions in combination with antifibrotic therapies.

## Materials and Methods

### Antibodies and reagents

In this study, the primary antibodies used were TGF-β (rabbit polyclonal antibody; Santa Cruz Biotechnology, Santa Cruz, CA), CD77 (rat monoclonal antibody; Abcam, Cambridge, UK), E-cadherin (mouse monoclonal antibody; BD Biotechnology, Research Triangle Park, NC), N-cadherin (mouse monoclonal antibody; BD Biotechnology), α-SMA (mouse monoclonal antibody; Sigma-Aldrich, St. Louis, MO), Phospho-PI3K p85/p55 (rabbit polyclonal antibody; Cell Signaling Technology, Beverley, CA), PI3K p85 (rabbit polyclonal antibody; Cell Signaling), Phospho-AKT (rabbit polyclonal antibody; Cell Signaling), AKT (rabbit polyclonal antibody; Cell Signaling), fibronectin (rabbit polyclonal antibody; Santa Cruz Biotechnology), type IV collagen (rabbit polyclonal antibody; Santa Cruz Biotechnology), β-actin (mouse monoclonal antibody; Sigma-Aldrich) and α-tubulin (mouse monoclonal antibody; Sigma-Aldrich). Secondary antibodies were horseradish peroxidase-conjugated goat anti-rabbit IgG antibody (Santa Cruz Biotechnology), goat anti-mouse IgG antibody (Santa Cruz Biotechnology), and Alexa Fluor 488 goat anti-rat IgG antibody (Life Technologies, Carlsbad, CA).

Detecting reagents used in this study were Gb3 (Matreya, Pleasant Gap, PA), lyso-Gb3 (Sigma-Aldrich), recombinant α-galactosidase A (rhα-Gal A; Fabrazyme [agalsidase β]; Genzyme Corp., Cambridge, MA), inhibitor of TGF-β receptor I (ALK5, SB525334; Calbiochem, Houston, TX), N-Dodecanoyl-NBD-ceramide trihexoside (Matreya), and SCDase (Sigma-Aldrich).

### Cell lines and treatments

The HK2 cell line (human proximal renal tubular epithelial cells) and SV40 MES 13 cell line (mouse renal glomerular mesangial cells) were purchased from the American Type Culture Collection (Manassas, VA). HK2 cells were maintained in keratinocyte serum-free medium supplemented with 0.05 mg/ml bovine pituitary extract, 5 ng/ml human recombinant epidermal growth factor, and 5 μg/ml gentamicin. SV40 MES 13 cells were maintained in a 3:1 mixture of Dulbecco’s modified Eagle’s medium/Ham’s F12 medium with supplemented 5% fetal bovine serum, 14 mM HEPES, and 100 U/ml penicillin/streptomycin. All cells were grown at 37°C in 5% CO_2_ in a humidified incubator.

After the cells were seeded into plates and grown to subconfluence, the culture medium was replaced with serum-free medium, and the cells were treated at the same time with Gb3 (30 μM and 45 μM) or lyso-Gb3 (200 nM and 400 nM) for 24 h. Then, the samples treated with reagents were maintained with a 1:2 ratio of serum and serum-free medium for 48 h. The cells were treated with Gb3 or lyso-Gb3 combined with α-gal A (10 μg/ml), TGF-β receptor inhibitor (SB 525334, 5 μM), or SCDase (10 μg/ml). None of the inhibitors was toxic at the doses used (evaluated by cell viability assay, MTS assay; Promega, Madison, WI).

### Protein isolation and immunoblotting

Sample collection and immunoblotting followed previously described methods [18]. Signals were detected using a West Save Gold immunoblotting detection kit (Ab Frontier, Seoul, Korea) and SuperSignal West Dura system (Thermo Fisher, Rockford, IL). The intensity of each band was determined using Fujifilm Multi Gauge (V3.0; Science Lab, Tokyo, Japan) following the manufacturer’s instructions.

### Fluorescent uptake of lipids and immunocytochemistry

1 × 10^5^ HK2 cells were plated for uptake analysis in cell culture chamber slides (SPL Life Sciences, Gyeonggi-do, Korea), and cell numbers were determined at different time points (0 h, 5 min, 24, 48, 72, and 96 h). Gb3 and SCDase were incubated for 16 h at 37°C, essentially as described by Ito et al [19]. The HK2 cells were treated with the fluorescent analog NBD Gb3 (30 μM) with or without SCDase (250 μU/ml), and the nuclei were counterstained with DAPI (Vector Laboratories, Inc. Burlingame, CA).

In addition, 1 × 10^5^ HK2 cells treated with α-gal A siRNA were seeded on cell culture chamber slides (SPL Life Sciences) and fixed with 4% paraformaldehyde in phosphate-buffered solution (PBS) for 1 h at room temperature. After blocking with 2% bovine serum albumin (BSA) in 0.1% Tris-buffered saline and Tween 20, the cells were incubated with the rat anti-human CD77/Gb3 monoclonal antibody (diluted 1:500), and then Alexa Fluor 488 goat anti-rat IgG secondary antibody (diluted 1:1000) in 1% BSA/PBS incubated for 1 h. All cells were counterstained with DAPI and observed by imaging with a fluorescence microscope Zeiss LSM 510 Meta (Zeiss, Oberkochen, Germany), using a 40 × objective.

### RNA interference

Knockdowns of human and mouse α-gal A were performed using ON-TARGET*plus* from Dharmacon (19 mer; Fisher Scientific) as indicated. The ID numbers for α-gal A, nontargeting, and GAPDH siRNAs were LQ-048870-01, L-012538-00, D-001810-02, D-001830-01, D-001830-02, and D-001810-01, respectively. Each siRNA kit contained silencing by SMARTpool of four individual siRNAs, and stock concentrations of siRNAs were made at 20 μM in sterile RNase-free water with siRNA buffer consisting of 60 mM KCl, 6 mM HEPES-pH 7.5, and 0.2 mM MgCl_2_. Reconstituted siRNA were placed on a shaker for 30 min at room temperature and then stored at –70°C.

Briefly, 3.5 × 10^5^ HK2 were plated in 60 mm dishes the evening before transfection, and transfection with α-gal A siRNAs (30 nM and 50 nM) was done using Lipofectamine RNAiMAX Reagent (Invitrogen, Carlsbad, CA) in accordance with the manufacturer’s protocol. Subsequently, cells were cultured for 72 h after transfection at 37°C and were harvested at the indicated days for TGF-β expression immunoblotting, Gb3 expression immunocytochemistry, and quantitative real-time polymerase chain reaction (PCR).

### RNA extraction

A total of 3.5 × 10^5^ HK2 per pellet were used for RNA extraction. On reaching confluency, the cultured cells were lysed using a scraper and centrifuged. Then, genomic DNA contained within the lysate was eliminated, and RNA was extracted from the lysate using the RNeasy mini kit (Qiagen Inc, Valencia, CA), in accordance with the manufacturer’s instructions.

### Statistical analysis

Data were analyzed with Prism software (GraphPad, La Jolla, CA) using *t*-test and analysis of variance (ANOVA), expressed as mean ± SEM. *P*-values less than 0.05 were considered to represent statistical significance.

## Results

### Exogenous fluorescent-labeled Gb3 treatment in HK2 cells

**Fig 1:**
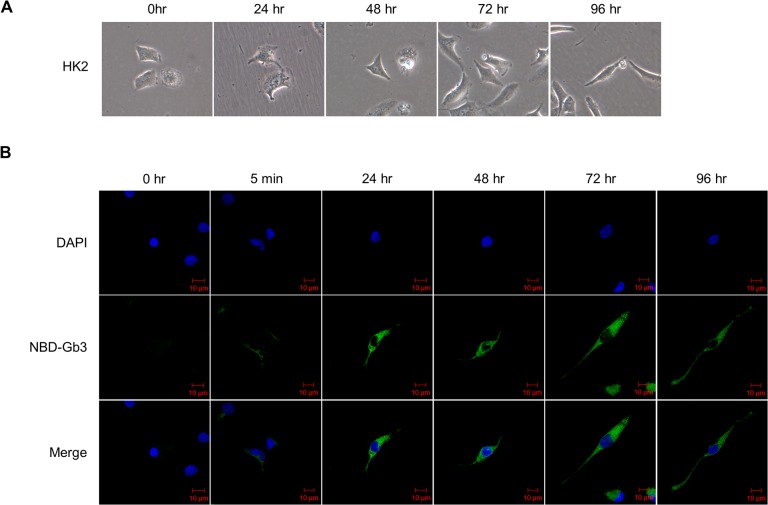
Fluorescent uptake analysis of Gb3 at indicated time points in HK2 cells. (**A**) Morphology of control HK2 cells cultured in complete media, imaged in time-dependent manner under the phase contrast microscope. (**B**) EMT morphological alteration using fluorescent analog Gb3 (in concentration of 30 μM) observed in time-dependent manner of 0 h to 96 h in HK2 cells. Fluorescent used is N-Dodecanoyl-NBD tagged Gb3 (green), and nuclei are stained with DAPI (blue). Fluorescent microscope observed using 40× objective. Scale bars, 10 μm.

Gb3 uptake was identified by treating HK2 cells with a medium added with a fluorescently-labeled Gb3 (N-Dodecanoyl-NBD-ceramide trihexoside) in which the fluorochromes were attached to acyl groups of Gb3 (Fig 1). Fluorescent Gb3 was weakly detected inside the cells 5 min after treatment, and persisted up to 96 h. In addition, HK2 cells underwent morphological changes over the course of time (0 h, 5 min, 24 h, 48 h, 72 h, and 96 h), and the cells adopted a more fibroblast-like morphology.

### Gb3 and lyso-Gb3 induced TGF-β dependent EMT

We initially determined the optimum concentrations and time required for Gb3 and lyso-Gb3 to initiate EMT in HK2 cells. The expressions of TGF-β, E-cadherin (a molecule that plays a key role in maintaining the integrity of epithelial cells and is regarded as an epithelial marker), N-cadherin (a mesenchymal marker), and α-SMA (a myofibroblast marker) were determined after Gb3 or lyso-Gb3 treatments.

**Fig 2:**
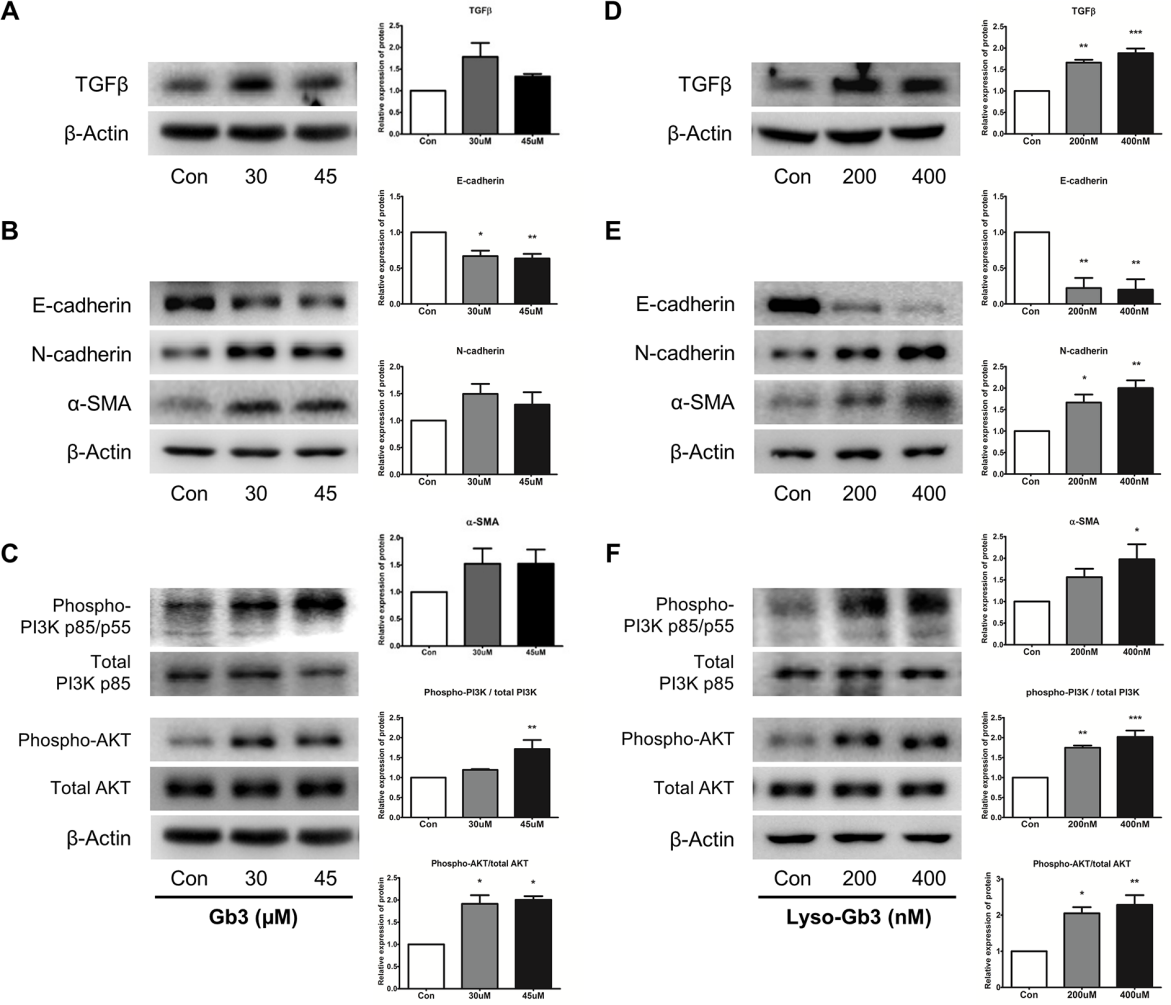
Effects of Gb3 and lyso-Gb3 treatments on EMT through PI3K/AKT pathway in HK2 cells. (**A–C**) Immunoblotting analysis of TGF-β, EMT markers, and phosphorylation of PI3K/AKT following Gb3 (30 μM and 45 μM) treatment of HK2 cells for 72 h. (**D–F**) Immunoblotting analysis of TGF-β, EMT markers, and phosphorylation of PI3K/AKT following lyso-Gb3 (200 nM and 400 nM) treatment of HK2 cells for 72 h. Panels (**A–F**) show a representative experiment (right panel), and data are presented as mean ± SEM, *n* = 3. Gb3 or lyso-Gb3 vs control, One-way ANOVA. * *P* < 0.05, ** *P* < 0.001, and *** *P* < 0.0001.

The TGF-β expression levels were increased when treated with Gb3 or lyso-Gb3 in 200 nM and 400 nM, compared with control HK2 cells (Fig 2A and 2D). In particular, upregulation of TGF-β was more typically observed after lyso-Gb3 treatment than Gb3 treatment. The expression levels of EMT markers in HK2 cells treated with Gb3 and lyso-Gb3 were also similar to the TGF-β expression pattern. The expression level of E-cadherin was significantly decreased by lyso-Gb3 treatment, whereas the expression levels of N-cadherin and α-SMA increased at concentrations of 200 nM and 400 nM. The expression levels of E-cadherin, N-cadherin, and α-SMA were changed by treatment with Gb3 at high concentrations, 30 μM and 45 μM; by contrast, the changes in the expression levels were markedly less than those of cells treated with lyso-Gb3 at low concentrations (Fig 2B and 2E).

### Gb3 and lyso-Gb3 treatments are associated with the activation of PI3K pathway

In this study, the expression levels of PI3K and AKT phosphorylation protein between control HK2 cells and HK2 cells treated with Gb3 or lyso-Gb3 were detected by immunoblotting. The activation of the PI3K/AKT pathway appears as a key feature of renal tubular EMT and renal fibrosis [20,21].

As shown in Fig 2C and 2F, phosphorylation of PI3K and AKT was moderate in control HK2 cells, but significantly increased in both Gb3- and lyso-Gb3-treated HK2 cells. These results showed that Gb3 and lyso-Gb3 activated phosphorylation of PI3K and AKT.

### Gb3 and lyso-Gb3 increases expression of ECM components

**Fig 3:**
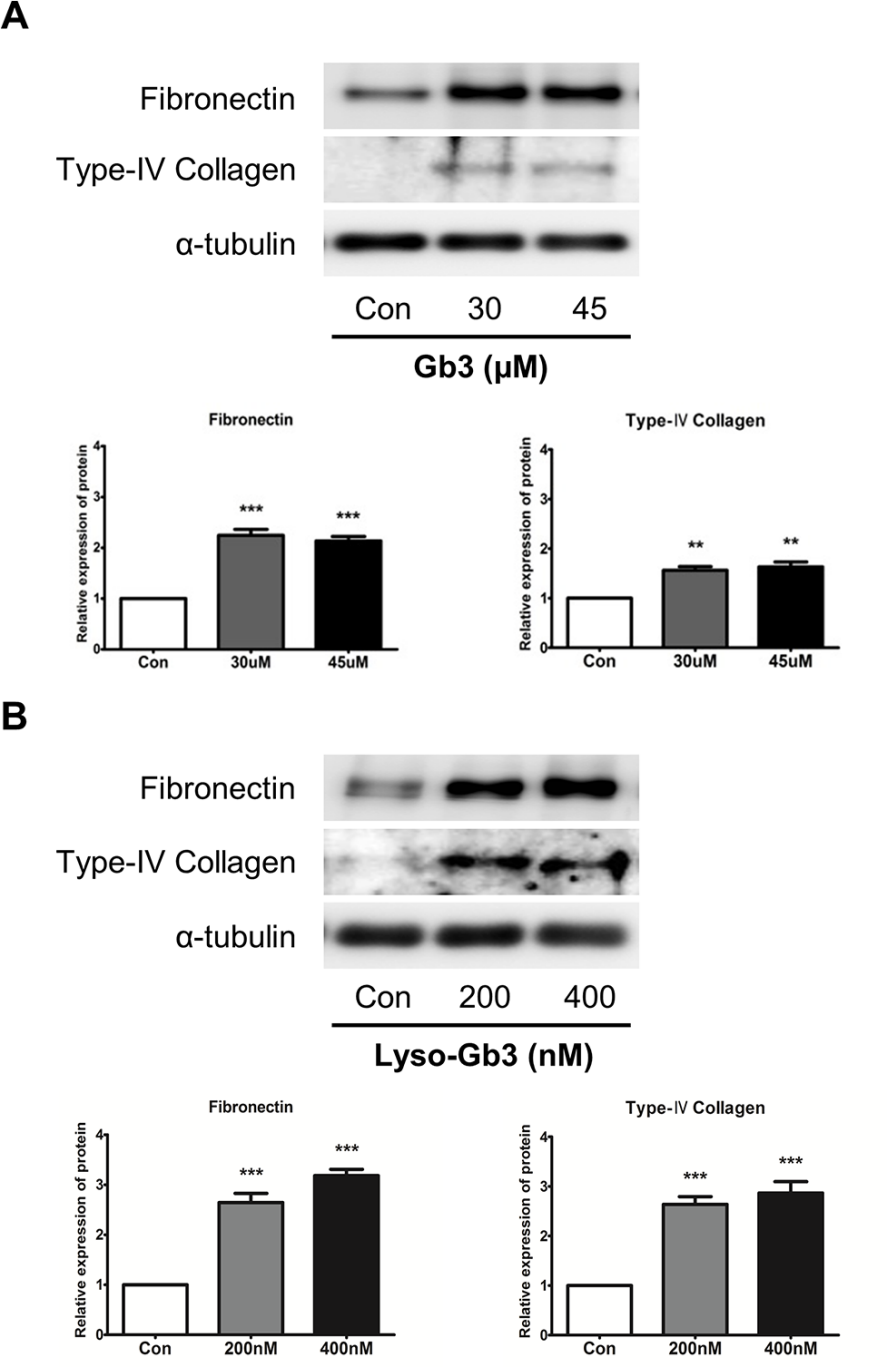
Effects of Gb3 and lyso-Gb3 treatments on ECM component expression in HK2 cells. (**A**) Immunoblotting analysis of fibronectin and type IV collagen following Gb3 (30 μM and 45 μM, respectively) treatment of HK2 cells for 72 h. (**B**) Immunoblotting analysis of fibronectin and type IV collagen following lyso-Gb3 (200 nM and 400 nM) treatment of HK2 cells for 72 h. Panels (**A**) and (**B**) show a representative experiment (bottom panel) and data as mean ± SEM, *n* = 3. Gb3 or lyso-Gb3 vs control, One-way ANOVA. * *P* < 0.05, ** *P* < 0.001, and *** *P* < 0.0001.

In HK2 cells, Gb3 and lyso-Gb3 increased protein levels of TGF-β, EMT marker, and PI3K/AKT phosphorylation. We performed immunoblotting analysis to confirm the fibrosis effect of Gb3 and lyso-Gb3. As shown in Fig 3A and 3B, the expression of ECM proteins such as fibronectin and type IV collagen was significantly increased by Gb3 and lyso-Gb3 treatments in HK2 cells. These results suggest that Gb3 and lyso-Gb3 could activate fibrosis progression by deposition of ECM components such as fibronectin, and collagen.

### Recombinant α-gal A administration prevents Gb3-induced EMT progress

**Fig 4:**
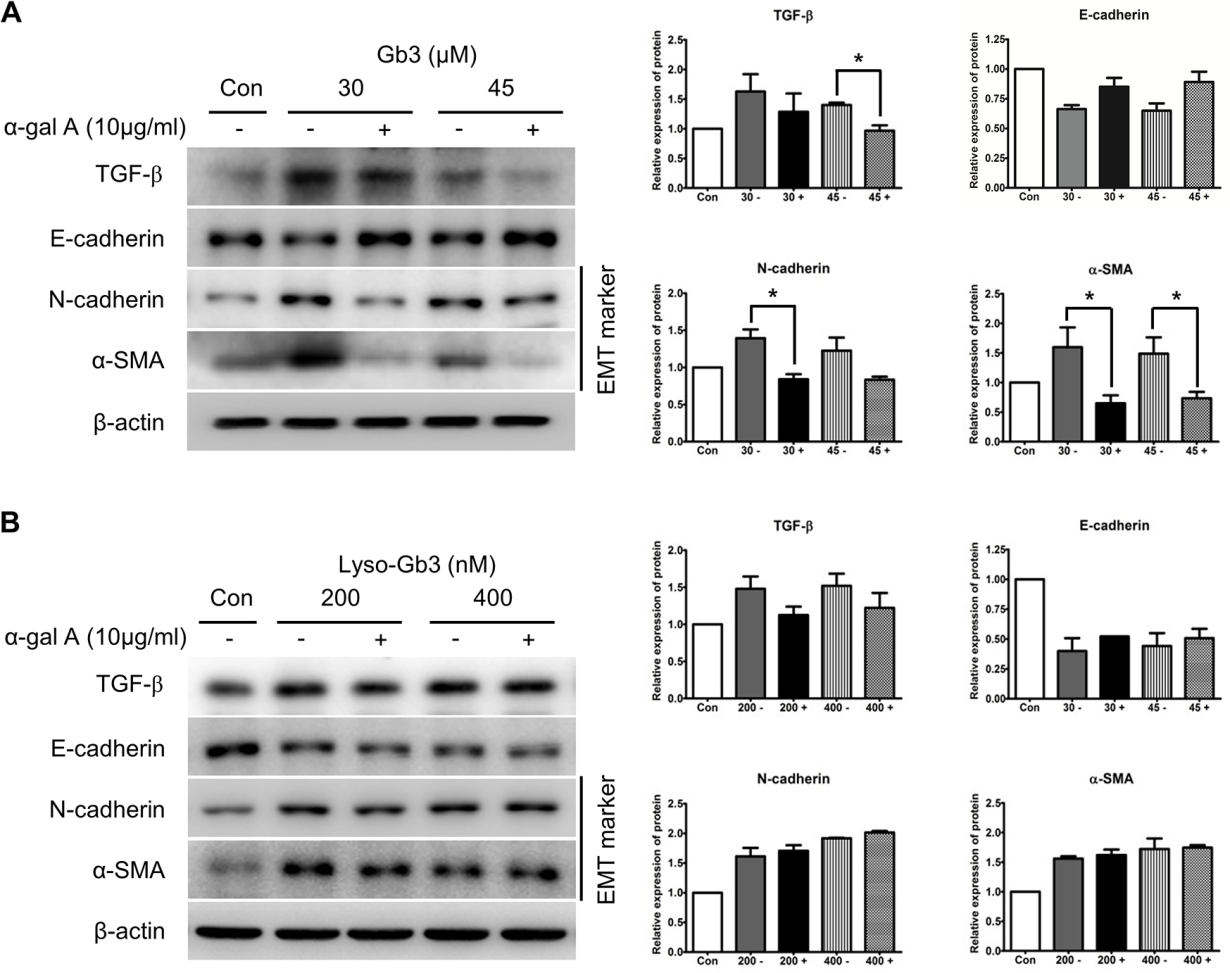
Effect of α-gal A on the expression of EMT markers induced by Gb3 and lyso-Gb3. (**A**) Immunoblotting analysis of expression of TGF-β and EMT markers induced by Gb3 (30 μM and 45 μM, respectively) in HK2 cells with or without α-gal A (10 μg/ml). (**B**) Immunoblotting analysis of expression of TGF-β and EMT markers induced by lyso-Gb3 (200 nM and 400 nM, respectively) in HK2 cells with or without α-gal A (10 μg/ml). Panels (**A**) and (**B**) show a representative experiment (right panel) and data as mean ± SEM, *n* = 3. (**A**) Gb3 vs Gb3 + α-gal A, (**B**) lyso-Gb3 vs lyso-Gb3 + α-gal A, *t*-test analysis. * *P* < 0.05, ** *P* < 0.001, and *** *P* < 0.0001.

The effect of α-gal A administration on Gb3 and lyso-Gb3-induced EMT was evaluated by checking the expressions of TGF-β and EMT markers (Fig 4A and 4B). Expression of TGF-β and EMT markers such as N-cadherin and α-SMA induced by Gb3, was inhibited by α-gal A treatment in HK2 cells (Fig 4A). However, α-gal A treatment did not inhibit the expression of TGF-β or EMT markers induced by lyso-Gb3 (Fig 4B). These results suggest that Gb3 induced EMT inhibited by the α-gal A, but lyso-Gb3 induced EMT is not inhibited by the α-gal A in HK2 cells.

### TGF-β receptor inhibition prevents EMT induced by Gb3 and lyso-Gb3

**Fig 5:**
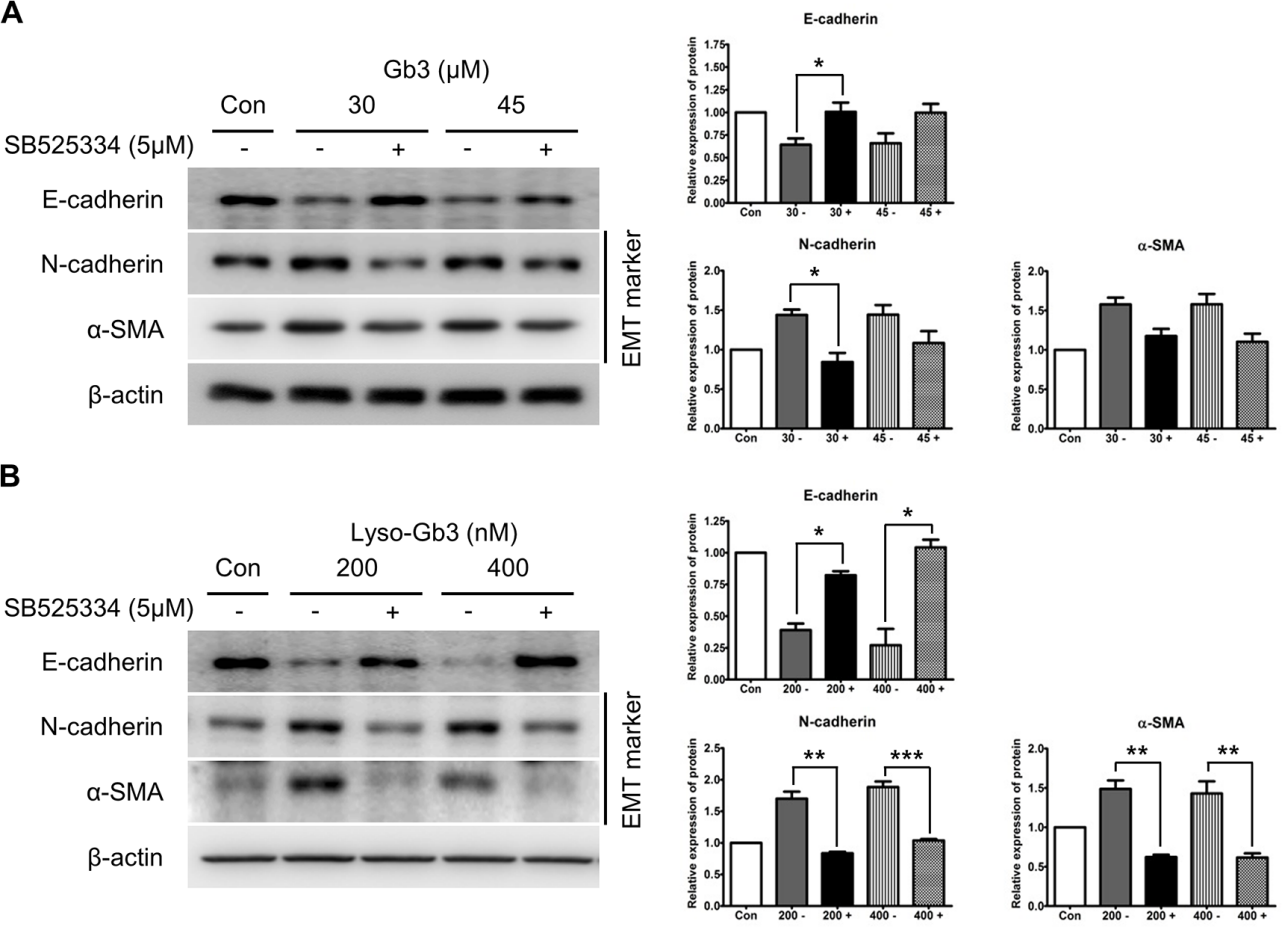
Effect of SB525334 on the expression of EMT markers induced by Gb3 and lyso-Gb3. (**A**) Immunoblotting analysis of expression of EMT markers induced by Gb3 in HK2 cells with or without SB525334 (30 μM and 45 μM, respectively). (**B**) Immunoblotting analysis of expression of EMT markers induced by lyso-Gb3 in HK2 cells with or without SB525334 (200 nM and 400 nM, respectively). Panels (**A**) and (**B**) show a representative experiment (right panel) and data as mean ± SEM, *n* = 3. (**A**) Gb3 vs Gb3 + SB525334, (**B**) lyso-Gb3 vs lyso-Gb3 + SB525334, *t*-test analysis. * *P* < 0.05, ** *P* < 0.001, and *** *P* < 0.0001.

To determine the potential treatment effectiveness alongside enzyme replacement therapy (ERT) and to clarify the role of the TGF-β pathway on EMT progression, we examined the blocking effect of TGF-β (TGF-β receptor inhibitor: SB 525334) in HK2 cells treated with Gb3 or lyso-Gb3 (Fig 5A and 5B). Blocking of TGF-β was shown to reverse the effect of Gb3 treatment and lyso-Gb3 treatment on the EMT markers. The blockage of TGF-β activated the expression of E-cadherin and inhibited the expression of EMT markers such as N-cadherin and α-SMA, and this effect differed significantly between SB525334-treated and untreated HK-2 cells in the lyso-Gb3-induced EMT (Fig 5B). The blockage of TGF-β also activated the expression of E-cadherin and inhibited the expressions of N-cadherin and α-SMA in Gb3-induced EMT. However, statistical significances were observed only at the changes of expression levels of E-cadherin and N-cadherin, both with 30 μM Gb3-treatment, between SB525334-treated and untreated HK-2 cells (Fig 5A).

### Lyso-Gb3 strongly induces the EMT response in HK2 cells

**Fig 6:**
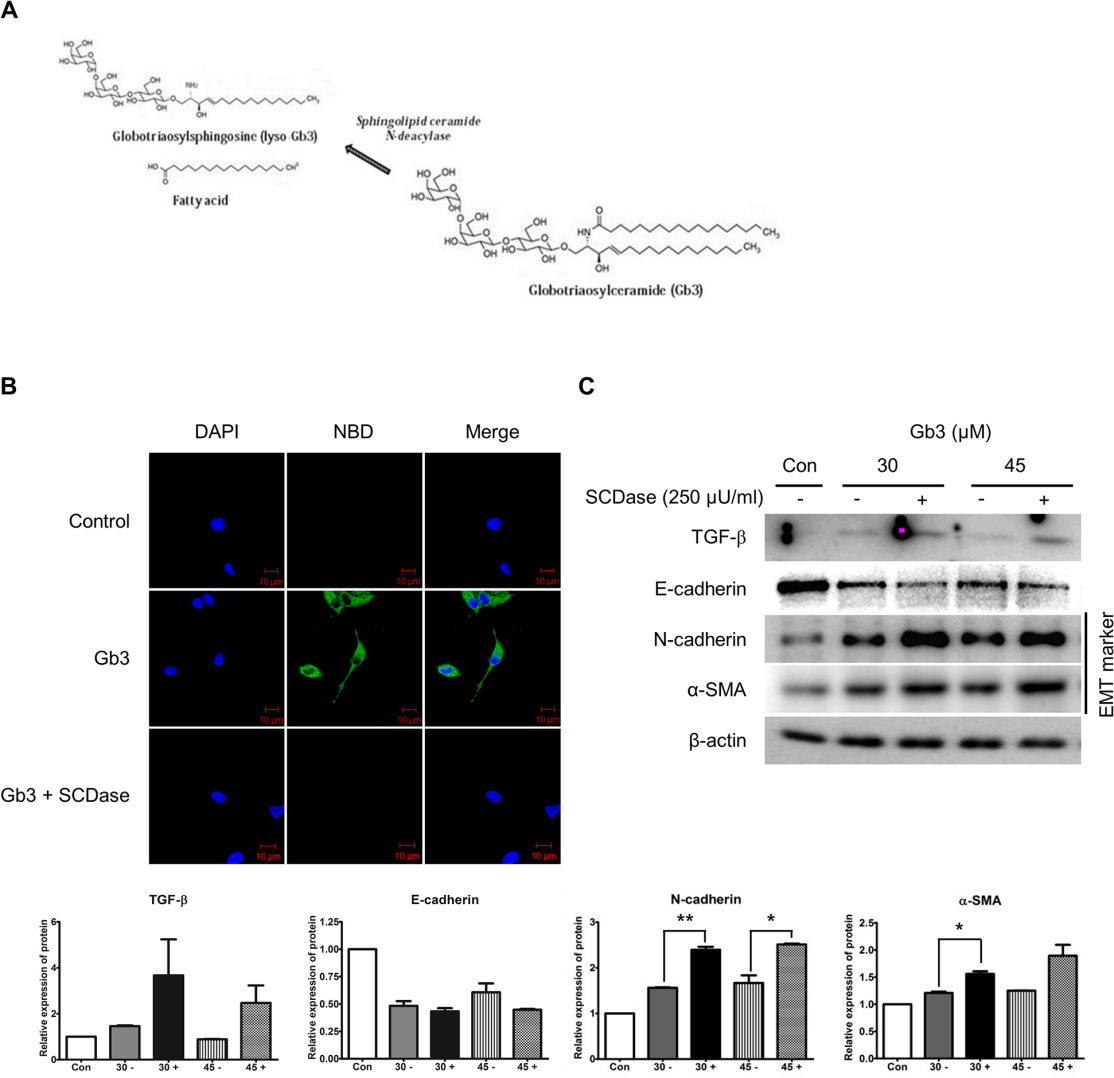
Effect of Gb3 hydrolyzed by SCDase on the expression of EMT markers by Gb3. (**A**) Chemical structure of Gb3 hydrolysis reaction catalyzed by SCDase. (**B**) Fluorescent labeled acyl group of Gb3 (N-Dodecanoyl NBD-Gb3, green staining) eliminated by SCDase. Fluorescent labeled Gb3 treated with SCDase (250 μU/ml) for 72 h in HK2 cells. The experiment was done by confocal microscopy and images were taken at 400× magnification. Scale bars, 10 μm. (**C**) The activity of TGF-β and EMT markers was assayed by the immunoblotting method treating of SCDase (250 μU/ml) and Gb3 (30 μM and 45 μM, respectively) for 72 h in HK2 cells. Panel (**C**) shows a representative experiment and data as mean ± SEM, *n* = 3. Gb3 vs Gb3 + SCDase, *t*-test analysis. * *P* < 0.05, ** *P* < 0.001, and *** *P* < 0.0001.

Sphingolipid ceramide *N*-deacylase (SCDase; Sigma) hydrolyzes the *N*-acyl linkage between fatty acids and sphingosine bases in ceramides of various sphingolipids [19]. Fig 6A shows the hydrolysis reaction of Gb3 being catalyzed by SCDase. To confirm the EMT effect of lyso-Gb3, we treated SCDase to convert Gb3 to lyso-Gb3. Conversion of Gb3 to lyso-Gb3 by SCDase was confirmed using the fluorescence analog labeled by the acyl group of Gb3 (NBD-Gb3). The fluorescence analog-labeled Gb3 was eliminated by SCDase treatment (Fig 6B).

The expressions of EMT markers induced by SCDase-treated Gb3 were confirmed by immunoblotting. The SCDase-treated Gb3 strongly stimulated the EMT phenomenon in HK2 cells (Fig 6C). The expression levels of N-cadherin and α-SMA induced by SCDase-treated Gb3 increased significantly more than those of the markers induced by Gb3. Although there was no statistical significance, the expression level of TGF-β was increased by SCDase-treated Gb3. These results suggest that lyso-Gb3, converted form Gb3 by SCDase, strongly induced EMT in HK2 cells.

### Knockdown of α-gal A mediates TGF-β-induced EMT

**Fig 7:**
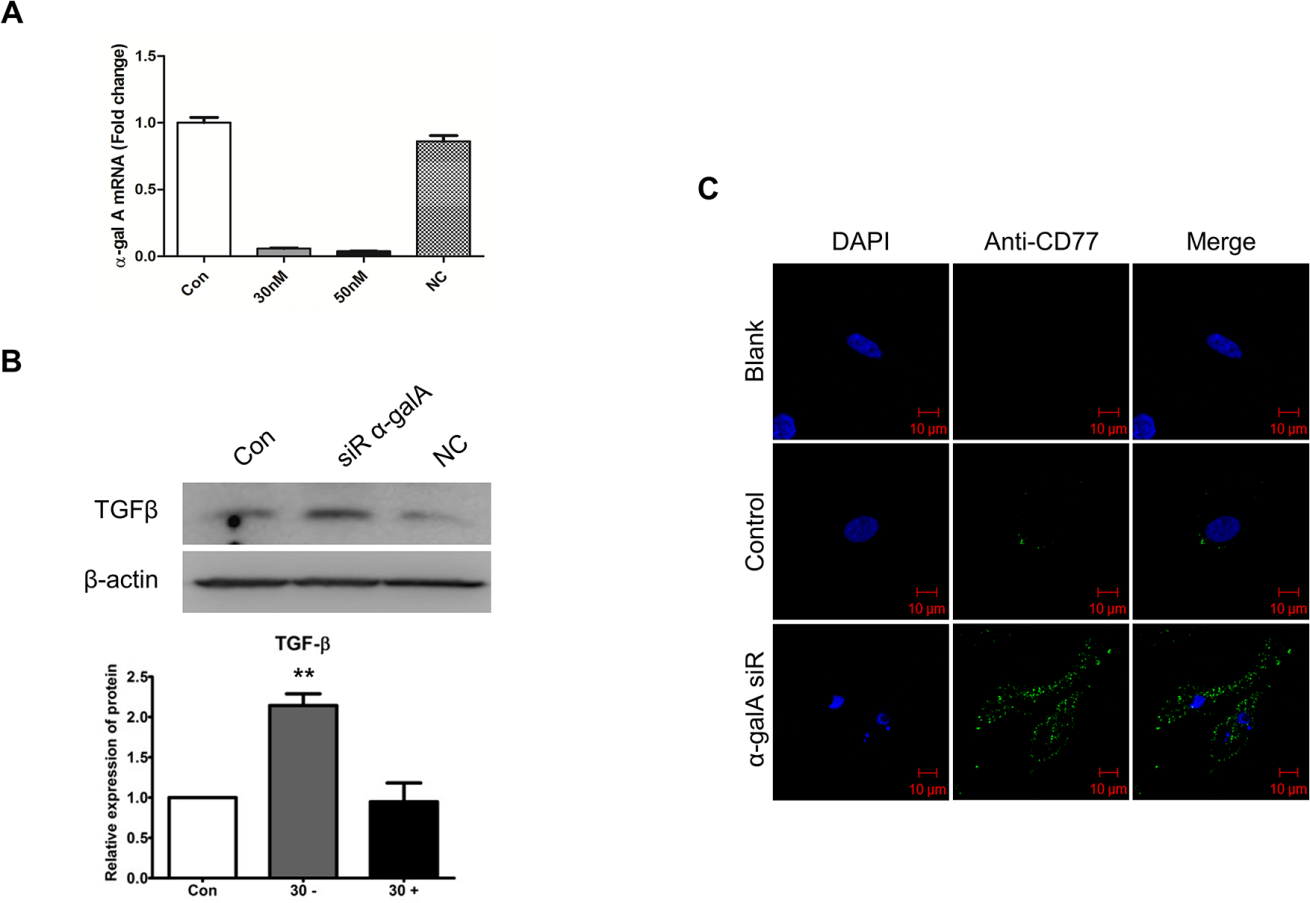
Effects of the expression of TGF-β and Gb3 after α-gal A silencing in HK2 cells. (**A**) HK2 cells were transfected with α-gal A-specific siRNAs (30 nM) for 72 h, and expression of α-gal A after siRNA transfection was tested using real-time PCR. Almost no α-gal A was detected in transfected cells. (**B**) Immunoblotting analysis of TGF-β expression in HK2 cells 3 days after α-gal A siRNA silencing. (**C**) Immunostaining of Gb3 in HK2 cells after α-gal A siRNA silencing. Fluorescence of Gb3 was detected by confocal microscopy with Alexa Fluor 488 IgG secondary antibody (green staining). Nuclei were stained with DAPI (blue). Fluorescent microscope observed using a 40 × objective. Scale bars, 10 μm. Panel (**B**) shows a representative experiment and data as mean ± SEM, *n* = 3. Control vs siR α-gal A, One-way ANOVA. * *P* < 0.05, ** *P* < 0.001, and *** *P* < 0.0001.

To determine whether silenced α-gal A, as an intracellular Gb3 accumulation model of FD, would induce TGF-β-mediated EMT, siRNA was used to silence α-gal A gene expression in HK2 cells. The interference efficiencies of siRNA were confirmed by real-time PCR and immunoblotting, and the transfection efficiencies were approximately 95% (Fig 7A). Immunoblotting analysis revealed that TGF-β protein expression was significantly increased by α-gal A gene silencing by siRNA, while pooled negative control siRNA treatment had no detectable effect on TGF-β protein expression (Fig 7B). Furthermore, immunocytochemistry for Gb3 (CD77) expression under conditions of α-gal A deficiency were examined (Fig 7C). The α-gal A-silenced cells significantly upregulated the expressions of CD77/Gb3.

### Phenotypic changes in kidney mesangial cells induced by Gb3 and lyso-Gb3

**Fig 8:**
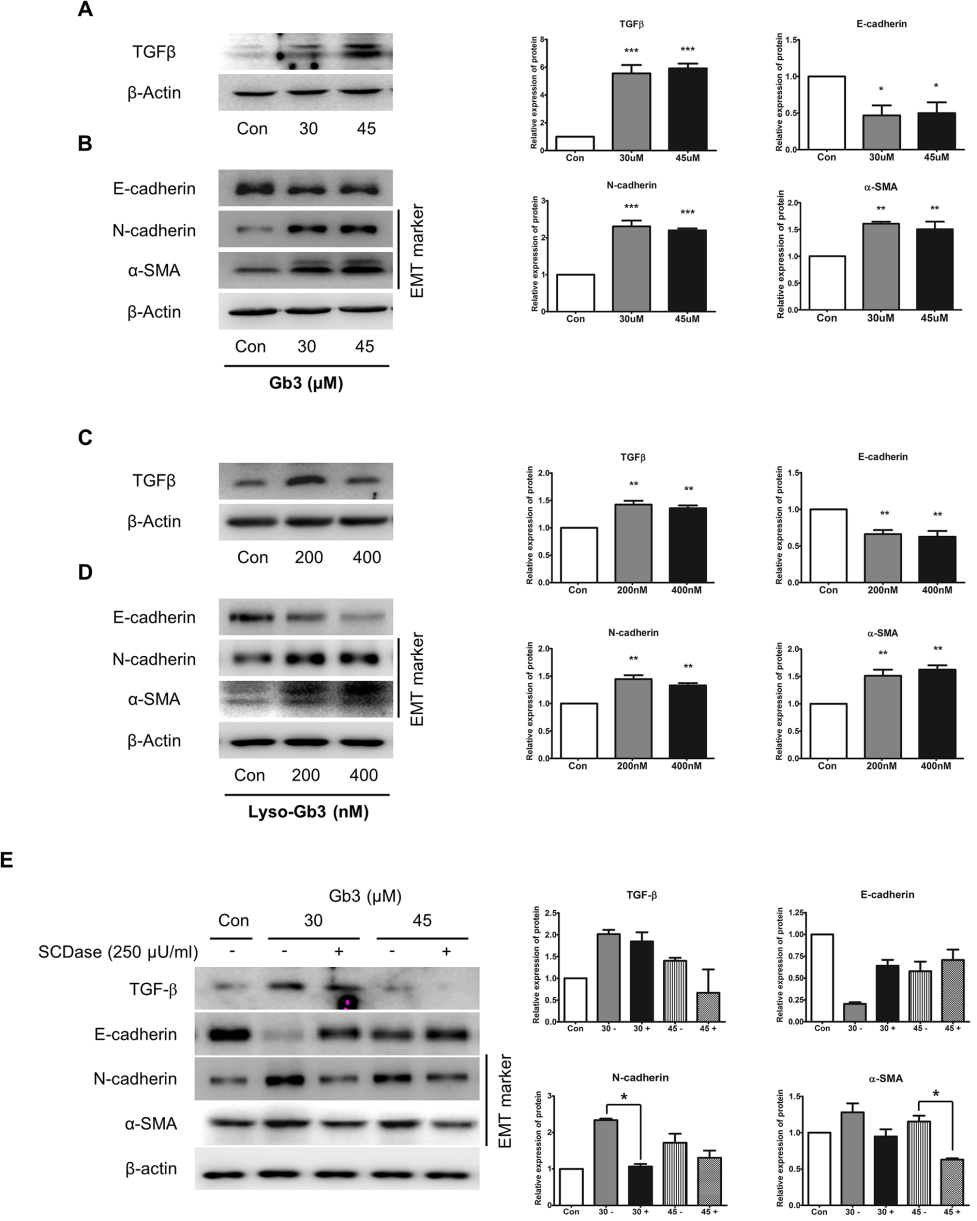
Effects of the expression of EMT markers by Gb3 and SCDase in SV40 MES 13 cells. (**A, B**) Immunoblotting analysis of TGF-β and EMT markers following Gb3 (30 μM and 45 μM) treatment of SV40 MES 13 cells for 72 h. (**C**, **D**) Immunoblotting analysis of TGF-β and EMT markers following lyso-Gb3 (200 nM and 400 nM) treatment of SV40 MES 13 cells for 72 h. (**E**) The activity of TGF-β and EMT markers was assayed by the immunoblotting method treating of SCDase (250 μU/ml) and Gb3 (30 μM and 45 μM, respectively) for 72 h in SV40 MES 13 cells. Panels (**A–E**) show a representative experiment (right panel) and data as mean ± SEM, *n* = 3. (**A–D**) Gb3 or lyso-Gb3 vs control, (**E**) Gb3 vs Gb3 + SCDase, One-way ANOVA. * *P* < 0.05, ** *P* < 0.001, and *** *P* < 0.0001.

The SV40 MES 13 cells, which are myofibroblast-like cells, also induced phenotype changes by Gb3 and lyso-Gb3. TGF-β expression, and the expression of EMT markers such as decrease of E-cadherin and increase of N-cadherin and α-SMA expression, were observed in cells treated with Gb3 or lyso-Gb3 (Fig 8A–8D). The expression levels of TGF-β, N-cadherin, and α-SMA induced by SCDase-treated Gb3 were reduced, and the expression level of E-cadherin induced by SCDase-treated Gb3 was increased in the SV40 MES 13 cells (Fig 8E). These results suggest that Gb3 strongly induced phenotype changes in SV40 MES 13 cells.

## Discussion

FD progresses to irreversible tissue damage and organ dysfunction by glycosphingolipid accumulation in lysosomes, extralysosomes, and extracellular space [3]. Among the complications of patients with FD, renal failure leads to significant morbidity and mortality [22]. In Fabry nephropathy, glycolipids are deposited in capillary renal cells (podocytes, tubular cells, glomerular endothelial, mesangial, and interstitial cells) [23]. Renal fibrosis is a key feature of Fabry nephropathy, but cellular and molecular mechanisms linking FD and renal fibrosis are not fully understood. Glomerulosclerosis and interstitial fibrosis are found in children with early stage tissue injury, along with features of podocyte injury, such as foot process effacement [24]. Glomerulosclerosis and interstitial fibrosis have also been observed in females with normal renal function and in the absence of overt proteinuria [25]. Based on these biopsy findings, Weidemann et al. suggested that early podocyte injury and fibrosis generated by epithelial cells increase as FD progresses [5]. In the present study, we focused on the EMT induction by Gb3 or lyso-Gb3 on tubular cells, not podocytes. We observed that Gb3 and lyso-Gb3 induce EMT, implying renal fibrosis, in HK2 cell lines. Before assessing the effect of EMT, we observed fibroblast-like morphology, such as an elongated phenotype and scattering of cells, characteristic of an EMT.

Our findings suggest that exogenous treatments of Gb3 and lyso-Gb3 are involved with various cellular mechanisms of EMT and ECM components, implying renal fibrosis, in tubular epithelial cells. In this study, proximal tubular cells and glomerular mesangial cells were used to find EMT by Gb3 or lyso-Gb3. The effect of lyso-Gb3 of low concentration was more significant than that of Gb3 in HK2 cells, including the downregulation of the epithelial marker E-cadherin, upregulation of mesenchymal markers N-cadherin, α-SMA, fibronectin, and type IV collagen, and a crucial mediator of ECM production, TGF-β. In a previous study, lyso-Gb3 activated TGF-β mRNA levels in a dose-dependent manner from 10 to 100 nM and the expression of ECM proteins, such as fibronectin and type IV collagen, in cultured human podocytes [15]. Like the results of HK2 cells, SV40 MES 13 cells, myofibroblast-like morphology, also were induced a phenotype change with the expression of TGF-β and EMT markers by Gb3 or lyso-Gb3.

We also confirmed the AKT/PI3K pathway was strongly activated in lyso-Gb3-treated HK2 cells. The PI3K/AKT pathway through TGF-β has been associated with EMT by its apparent regulation of processes such as cytoskeleton organization, cell growth, survival, migration, and invasion [13,26]. These results suggest that Gb3 and lyso-Gb3 induce EMT by activation of PI3K/AKT signaling through upregulation of TGF-β in HK2 cells. Furthermore, lyso-Gb3, but not Gb3, might have a strong relationship with renal fibrosis through phosphorylation of PI3K/AKT expressed at a low concentration.

ERT is the most fundamental management tool used for the treatment of FD. However, ERT has limitations; it is less effective when the disease has progressed as far as kidney injury and renal fibrosis [27,28]. Also, plasma lyso-Gb3 is increased in classically affected Fabry patients, and is reduced but not normalized following ERT of recombinant α-gal A, while it is undetectable in normal human plasma [4,29,30]. This limitation was confirmed in our experiments. Our investigation has indicated that treatment of α-gal A can reduce Gb3-induced EMT, but does not normalize lyso-Gb3-induced EMT.

Therefore, new therapeutic approaches based on a better understanding of pathogenic factors are needed to supplement ERT to improve treatment outcomes. We tried to find novel therapeutic possibilities using SB525334, a potent and selective inhibitor of the TGF-β receptor I (ALK5), as an anti-EMT drug in HK2 cells, in addition to α-gal A ERT for FD patients. Interestingly, the treatment with SB525334 inhibits the expression of EMT markers by Gb3 and lyso-Gb3. These findings indicate that a more effective potential treatment may be possible by using SB525334 alongside ERT, which extends the therapeutic limits of ongoing FD. Furthermore, prevention of EMT expression by SB525334 was observed more typically in lyso-Gb3-treated cells than Gb3-treated cells. Taken together with EMT induction by Gb3 or lyso-Gb3 in HK2 cells, these results suggest that Gb3 and lyso-Gb3 may contribute to the progress of renal fibrosis in FD by cell-specific features.

To observe the cell-specific expressions of EMT markers induced by Gb3 or lyso-Gb3 in kidney cells, we used SCDase to convert Gb3 into lyso-Gb3 in culture media. The lyso-Gb3 produced by cutting the fluorescent analog attached to the acyl group of Gb3 differentially regulated the expression of EMT markers in each kidney cell type. The lyso-Gb3 that was converted by SCDase treatment induced the EMT of HK2 cells, as did direct exposure of lyso-Gb3 to culture medium. However, it induced the downregulation of EMT markers in SV40 MES 13 cells. These results strongly suggest that renal tubular epithelial cells are more susceptible to lyso-Gb3, but mesangial cells are more susceptible to Gb3.

Our study used extracellular Gb3 treatment onto cultured HK2 cells. We tried to determine whether intracellular accumulation of Gb3 can also induce EMT in HK2 cells. Consistent with a previous study [31], we observed that the Gb3 expression was upregulated when the expression of α-gal A was blocked. We also observed that α-gal A deficiency induced the upregulation of TGF-β expressions. The EMT can be induced in these α-gal A deficient cells through upregulation of TGF-β expressions.

Among studies to understand questions such as the primary causes and risk factors of FD, the cellular and molecular mechanisms, and the potential therapeutic approach of renal fibrosis in Fabry nephropathy, lyso-Gb3 has been suggested to be a promoter of the renal fibrosis caused by FD. Lyso-Gb3 is known to be a water-soluble compound with properties that could be easily moved in and out of cells, and in some ways, it may behave as an accumulated soluble mediator, leading to tissue injury [4]. The increased plasma lyso-Gb3 in patients with FD may lead to EMT in particular kidney cells, such as kidney tubular cells, as well as podocytes [5,15].

Consistent with previous reports [15,30], our study demonstrated that extracellular as well as intracellular Gb3 and lyso-Gb3 can potentiate EMT processes via the TGF-β/AKT/PI3K signaling pathway in tubular epithelial cells, and suggests that cell- and substrate-specific induction of EMT by Gb3 and lyso-Gb3 on kidney cells can contribute to disease progression after initial podocyte injury. This study also suggests that anti-EMT drug developments such as TGF-β inhibitors, in addition to ERT, can provide better therapeutic options for FD patients.
